# Communication Between Anaesthesia Providers for Clinical and Professional Purposes: A Scoping Review

**DOI:** 10.1155/anrp/3598234

**Published:** 2025-03-06

**Authors:** Hilary Edgcombe, Gatwiri Murithi, Mudola Manyano, Sophie Dunin, Neal Thurley, Helen Higham, Mike English, Claire Blacklock

**Affiliations:** ^1^Health Systems Collaborative, Nuffield Department of Medicine, University of Oxford, Oxford, UK; ^2^Center for Equity in Global Surgery, University of Global Health Equity, Kigali, Rwanda; ^3^Department of Anaesthesia, North Cumbria Integrated Care NHS Foundation Trust, Cumbria, UK; ^4^Nuffield Department of Anaesthetics, Oxford University Hospitals NHS Foundation Trust, Oxford, UK; ^5^Bodleian Health Care Libraries, University of Oxford, Oxford, UK; ^6^Nuffield Department of Anaesthetics, University of Oxford, Oxford, UK

## Abstract

**Background:** Anaesthesia providers in all contexts need to be able to communicate with colleagues to meet a variety of clinical and professional needs, including physical help, advice and support as well as learning, supervision and mentorship. Such communication can be regarded as a ‘social resource' which underpins anaesthesia providers' practice, but which has not itself been extensively studied. The objective of this scoping review is to provide an overview of the literature related to communication among anaesthesia providers to meet clinical and professional goals, focusing on the modalities, contexts and purposes or outcomes of such communication, as well as which providers are involved.

**Methods:** We conducted a scoping review using the JBI methodology to examine the current literature available, searching the Cochrane Database of Systematic Reviews and Cochrane Central Register of Controlled Trials, Medline, Embase, CINAHL and Google Scholar. Papers were eligible for inclusion where they primarily addressed the subject of communication between trained anaesthesia providers for any clinical or professional purpose (excluding purely social interactions). Data were charted for the location and cadre of providers represented, means of communication and the situation, purposes and outcomes of communication.

**Results:** 3872 records were identified for screening, and 225 papers were ultimately included. Communication was reported both as a variable influencing a wide range of clinical and nonclinical outcomes and as an outcome in itself which might be modified by other factors. It was also considered in a smaller group of studies as a resource with varying availability to anaesthesia providers. Physician providers were well represented in included documents, but nurse anaesthetists, clinical officers and other nonphysician, nonnurse anaesthetists were far less commonly included. The majority of identified studies on communication between anaesthesia providers originated from and related to high-income countries.

**Conclusion:** Communication between anaesthesia providers affects all aspects of their practice and has implications for both patient outcomes and workforce capacity. More research is necessary to understand how the availability of communication as a resource affects patient care and health worker well-being, particularly in low- and middle-income contexts and among nonphysician anaesthesia providers.

## 1. Introduction

The global anaesthesia workforce consists of physician and nonphysician providers with heterogeneous training backgrounds who work in a wide variety of contexts [1, 2]. Nonetheless, common to every anaesthesia provider's (AP's) experience is the aim to provide safe, high-quality anaesthesia. Considerable attention has been given to the physical and pharmaceutical resources required by anaesthetists to do so, in both high- and low-income contexts [3, 4]. Relatively little attention has focused on how APs draw on social resource to support their clinical and professional work, despite work in wider healthcare contexts suggesting the relevance of social interaction to both patient safety [5] and workforce well-being [6]. It is the aim of this scoping review to describe and map the existing literature on how APs communicate with one another in a variety of contexts, i.e., how they access social resource to support their practice.

The practice of safe anaesthesia requires the anaesthetist to be well informed, up to date in professional developments, know their capability and enlist help beyond it, anticipate and recognise complications emergently, have the capacity to treat those complications and maintain their own well-being [7, 8]. All these presume the availability of a professional social resource, i.e., access to (formal or informal) interaction with other APs, to provide professional and clinical support as needed. However, the nature of anaesthetic practice, especially in resource limited settings, means that many providers work as the sole representative of their speciality in any given operating theatre. The nearest colleague may be in the same theatre suite, in the same hospital or geographically distant. Different health system contexts and regions have different structures and systems which influence the ability and access of anaesthetists to communicate with colleagues.

Globally, independent anaesthesia care is delivered by more than one cadre of provider. In high-income countries, the predominant providers are physicians who have undertaken specialist postgraduate training (67%) [9]. Low- and middle-income countries (LMICs) tend to rely more heavily on nonphysician anaesthesia providers (NPAPs) (in low-income countries, NPAPs constitute 86% of all APs [9]) even in areas where physician training in anaesthesia exists [2]. In rural or remote district hospitals in LMICs, an NPAP may work alone under significant resource constraints, managing patients from even more remote areas with advanced and/or emergency surgical conditions [3]. It is likely that social resource is more limited for NPAPs working in rural LMIC contexts than for PAPs in HICs; nonetheless, the functional interaction of providers can be seen as a ‘software component' [10] crucial to the effective function of the complex healthcare system required to provide safe surgery whatever the context.

While the value of social resource in anaesthesia is well recognised at an experiential and anecdotal level by APs, relatively little and disparate literature exists in this area. This scoping review aims to discover what evidence exists related to communication between APs, including but not limited to: how, when and why APs communicate, the outcomes of their communication and means of optimising effective communication. A scoping review methodological approach was deemed appropriate for investigating this topic in light of the anticipated heterogeneity of evidence around inter-AP communication including the breadth of situations in which communication might be a primary theme and the range of conceptual and methodological approaches which might be employed.

### 1.1. Key Elements of the Search Strategy

Preliminary searches demonstrated that searching based on the two categories ‘anaesthesia providers' and ‘communication' (+ related terms) retrieved an unfeasibly large number of irrelevant publications. We therefore used our existing knowledge of the literature and further preliminary searches to identify concepts with direct applicability to health professionals' communication which could be used to develop a feasible and relevant search strategy. We identified three key concepts within healthcare literature which are closely related to inter-provider communication, which informed the conduct of our review.

The first is that of ‘communities of practice' (COPs). COPs have been defined as:

‘groups of people who share a concern, a set of problems or passion about a topic, and who deepen their knowledge and expertise in this area by interacting on an ongoing basis' [11].

At least in theory, COPs may contribute to several desirable outcomes for their members, including work satisfaction, motivation, high performance and safer organisations (and also may have negative effects) [12]. They may have arisen ‘organically' or have been intentionally developed and include virtual and geographically separated memberships. In the wider healthcare literature, much of the work on COPs has been oriented towards learning and development, with a more recent focus on implementation of evidence-based practice [13]. Many APs are likely to be members of formal and/or informal COPs.

The second key concept is that of ‘interprofessional practice'. Although, from a health system perspective, APs have similar *functional* identities, within the category of APs, providers vary in their *professional* identity, as physicians, nurses, clinical officers, midwives and pharmacists, and others may undertake different programmes of anaesthesia training. The most common framing of this kind of interprofessional practice in the context of anaesthesia is *task-sharing* (TS), an evolution of the ‘task-shifting' paradigm common in global health discourse [14]. A recent Delphi study defined TS as occurring:

‘when tasks are completed collaboratively between providers with different levels of training' [15].

Two recent reviews of TS in anaesthesia and surgery described ‘the tasks shifted, the health workers involved in TS and the role of supervision in TS' [16]; a review by Ashengo et al. on the same topic described data on the scope of tasks shared, training programs, effects on quality of care, acceptability and cost/cost-effectiveness as well as barriers to safe and effective TS [17]. Both reviews provide important overviews of key aspects of TS but do not explicitly examine communication within or outside this framework.

The third key concept used is that of 'networks'. Networks and COPs may examine similar phenomena through different lenses; where the literature on COPs orients towards shared professional identity and purpose, networks are often studied from a more structural perspective, focusing on the patterns of interaction between a variety of individuals. Interpersonal networks in social and healthcare contexts have been widely studied and conceptualised [18, 19]. Definitions vary in formality but at a minimum involve interactions between more than two individuals and generally imply a degree of stability (i.e., the chance of repeated interactions involving the same individuals). A review describing key features of effective professional networks in healthcare using social network analysis techniques identified that the structure of networks as well as the characteristics of network members is likely to be informative, but that the link between network structure and patient outcomes is still unclear [20].

These concepts may all in different ways be applied to APs and the ways in which they communicate for clinical and professional purposes. We therefore used the key concepts of COPs, interprofessional practice (plus its subsidiaries including TS and task-shifting) and networks to define our search strategy.

Communication undertaken by APs does not only occur with other APs; we note that good anaesthetist–patient communication, as well as effective communications within the wider surgical team, is fundamental to high-quality clinical care. Both have rightly received increasing attention in recent years particularly related to the central role of human factors in mediating patient safety [21–23]. However, these aspects of professional communication fall outside the scope of this review, which is specific to communication occurring between anaesthesia professionals, also critical for the safe provision of anaesthesia.

### 1.2. Objective and Review Questions

The objective of this scoping review is to map the available evidence regarding communication among APs to meet clinical and professional goals, in terms of the modalities, contexts and purposes/outcomes of inter-AP communication, as well as which providers are involved.

The overall review question is: What evidence exists on how APs communicate with one another to meet clinical and professional goals?

Subquestions:

a. Which groups of APs have been included in the literature on AP communication?
b. What modalities of inter-AP communication have been studied?
c. In what contexts has inter-AP communication been studied?
d. What purposes or outcomes of inter-AP communication have been studied?

PRISMA-ScR guidance was followed for this scoping review, and the PRISMA checklist is available as Supporting file 1: PRISMA-ScR Checklist.

## 2. Methods

### 2.1. Search Strategy

Searches were undertaken by a specialist librarian (NT) who searched four databases from inception to 30^th^ September 2022: the Cochrane Database of Systematic Reviews and Cochrane Central Register of Controlled Trials (Cochrane Library, Wiley) (Issue 9 of 12, September 2022), Medline (OvidSP) (1946–present), Embase (OvidSP) (1974–present) and CINAHL (EBSCO) (1981–present).

The searches comprised title/abstract keywords and subject headings for anaesthetists, communication and the key concepts: COPs, interprofessional practice and interpersonal networks. No date or language limits were applied at this stage. Google Scholar was also searched using a strategy based on the database searches and the first 150 results were retrieved for screening (an example of the full electronic search strategy is provided in Supporting file 2: Example search strategy). After screening, all included full text articles underwent reference list and citation searching for any additional relevant articles (HE).

Following analysis of the primary searches above, as this had taken some time, the four database searches were repeated by the primary author (HE) using the same search strategy, to 23^rd^ October 2024, in order to identify recently published relevant work. Articles retrieved in this latter search were screened and analysed by HE, using the same criteria as for the original set.

### 2.2. Source Selection

Eligibility criteria for inclusion of publications by participants, concept and context at screening are summarised in Table 1. All search results were deduplicated using EndNote: articles that were obviously non-English, non-French were discarded, reflecting the language capabilities of the reviewing team. A pilot test of inter-reviewer consistency was undertaken: titles and abstracts of 100 articles were screened by all five reviewers blinded to each other's results. The mean percentage agreement across all five reviewers was 86.4%. The mean prevalence-adjusted, bias-adjusted kappa (PABAK) for all ten possible reviewer pairs was 0.728, suggesting substantial agreement [24].

Title/abstract screening was, therefore, undertaken for all remaining articles by two independent reviewers using Rayyan.ai. Articles accepted via title-abstract screening were then screened again in full text by pairs of independent reviewers. Where there was disagreement about inclusion between paired reviewers at either the title-abstract or full-text screening stage, this was resolved by either discussion between the pair or a third reviewer's assessment. Further articles with full text not in English or French were excluded at the screening stage.

### 2.3. Data Charting

Abstracted data were transferred to an Excel spreadsheet from included papers (Supporting file 3: Data charting tool). 20% of the papers were charted by two reviewers independently (SD and HE) to confirm consistency of data collection and identify any iteration of the data charting tool which was required. A category was added at this stage (‘Place of communication in study') and retrospectively applied to retrieve data from the already charted papers. Analysis was undertaken by HE including the application of proposed analytic categories (operational level, domain and specific purposes) to the charted data. Themes and data were presented to and discussed with the research team to identify points of resonance, key concepts and gaps in the existing research as well as the likely implications for clinical, research and patient communities. We grouped studies by theme as well as by the criteria outlined in the review objectives.

## 3. Results

### 3.1. Characteristics of Retrieved Papers

**Figure 1:**
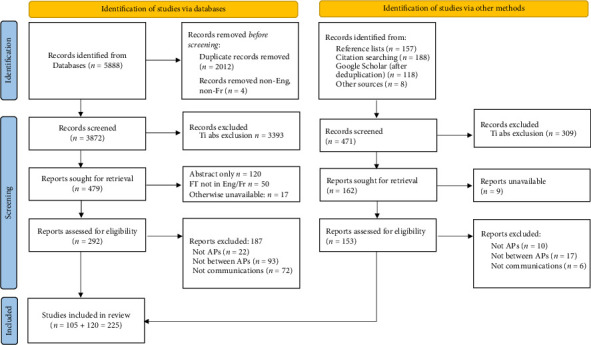
PRISMA diagram showing the identification and selection of papers for review.

Database searching yielded 5888 records, resolving to 3872 records for screening after deduplication; searches from other sources (reference lists, citation screening and Google Scholar) yielded an additional 471 records for screening. After title-abstract screening, a total of 641 records were sought for full text retrieval of which 225 were ultimately included for data extraction and analysis. Theses and published abstracts without further detail were excluded. Details are shown in the accompanying PRISMA diagram (Figure 1). Of the retrieved papers, 137/225 described original research (of which 98 were quantitative studies, 21 were qualitative studies and 18 had a mixed methods approach). The remaining 88 papers were reviews, letters, editorials or simple descriptions of a new technology or process. A full citation list of included papers is available as Supporting file 4: Citation list of included sources.

### 3.2. Overall Themes

This scoping exercise demonstrated the different perspectives with which authors view the topic of communication between APs. We identified two dominant approaches to examining communication:

1. ‘Communication as variable': These papers typically identify outcomes of importance (such as patient mortality [25], or AP burnout scores [26]), treating communication in some form as the/a significant variable which could influence the outcome of interest.
2. ‘Communication as outcome': This body of work includes studies which aim to evaluate how a particular approach might affect or improve the quality of communication in some way (for example, how interpersonal behaviour influences a trainee's ability to challenge a senior [27]). A substantial subgroup within this category is dedicated to examining specific technological or system modalities by which communication occurs (for example, the latency of a pager system [28], the speed of accessing emergency help with a ‘traffic light' verbal model [29]).

Both approaches are exemplified in the substantial body of work identified within this review which examines anaesthesia handovers: Some studies treat handover as the communication variable which may or may not influence clinical outcomes [25, 30], whereas other studies sought to improve communication at handover as an outcome by, e.g., introducing a new handover cognitive aid [31, 32].

A third, less frequent approach, treats ‘communication as resource'. Work using this approach identifies access to communication as the focus and studies evaluate ways of improving APs' access to communication with one another, using digital and other modalities [33, 34].

### 3.3. Representation of AP Groups

**Figure 2:**
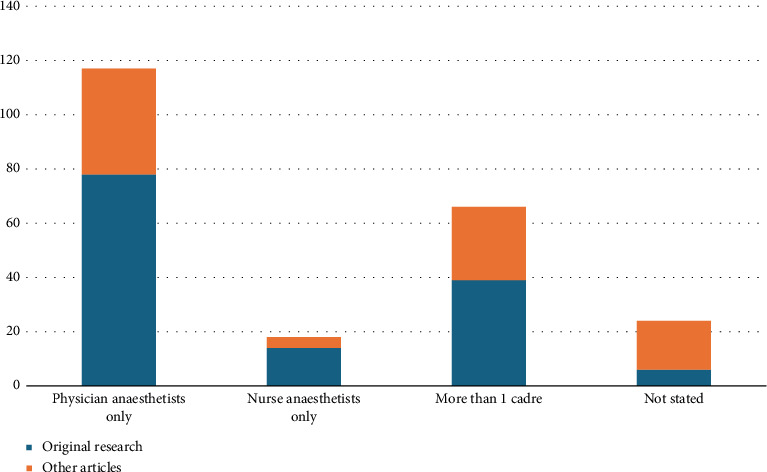
Bar graph showing the representation of provider groups in reviewed papers.

A variety of provider groups were studied in the literature retrieved, shown in Figure 2. Physicians (only or alongside other cadres) were the predominant group represented followed by nurse anaesthetists. Nonphysician, nonnurse anaesthetists (such as clinical officers) were considered explicitly in eight papers alongside physicians and/or nurses and as part of the general global workforce in seven further papers.

**Figure 3:**
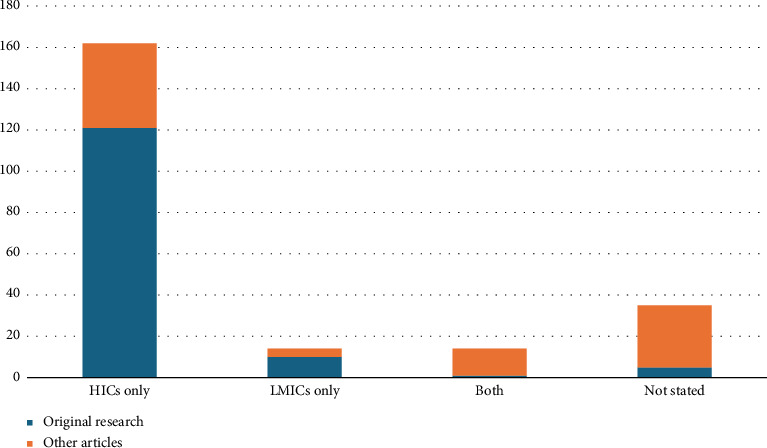
Bar graph showing the HIC/LMIC location of providers in reviewed papers.

**Figure 4:**
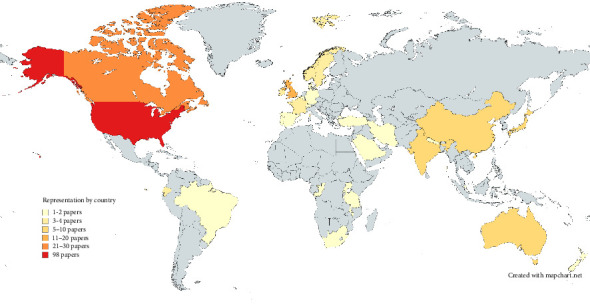
Map showing the country location of providers in reviewed papers⁣^∗^. ⁣^∗^excludes papers with generic scope of “Europe” (4), “LMICs” (3), “Global” (25) or “Unstated” (10).

162 (72%) of the included studies referred to anaesthetists in high-income countries alone, versus 14 (6.2%) addressing anaesthesia provision in LMICs alone (Figures 3 and 4). 14 articles (6.2%) included APs from both specific HICs and LMICs. Of these, six described medium-long term partnerships between APs in different countries, two described interaction between APs in different countries during a clinical case and the remaining articles discussed communication occurring within countries, but included more than one country in their scope. 35 articles did not state the location of APs considered or had an explicitly global scope.

### 3.4. Communication Modalities

**Figure 5:**
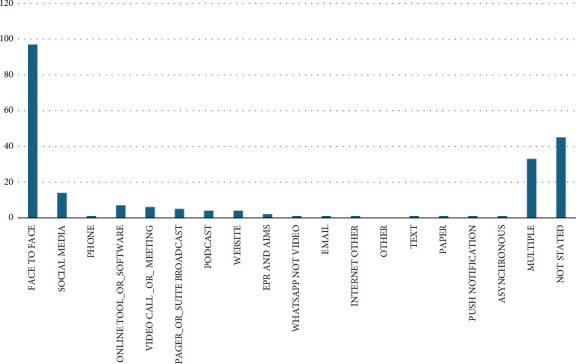
Bar graph showing the communication modalities represented in reviewed papers.

Communication modalities, where stated, included face to face communication, phone calls and several digital technology-based modalities (email, online messaging, social media, website-mediated communication and other software tools), shown in Figure 5. The majority of studies examined communication between two individuals only or between a small group of individuals known to one another. A small minority of studies addressed impersonal or ‘one to many' communication such as podcasts or YouTube.

### 3.5. Contexts and Purposes/Outcomes of Communication

We extracted and categorised data according to two related concepts: (1) the domain (clinical or professional context) within which communication was addressed and (2) the specific purpose of communication (where stated). The purpose here is understood as the reason for, justification of or motivation for communication.

Table 2 shows the scope of domains and purposes for which communication between providers was studied. These have been grouped into three categories. Where articles overlapped categories, their allocation was based on the category they most prominently featured.

1. ‘Achieving clinical output' which includes studies focused on the delivery of clinical care and organisational/institutional processes supporting this delivery
2. ‘Formal professional activity' which includes work related to the training and supervision of APs, evaluation of providers' activity and articles related to professional networks and collaborations
3. ‘Personal well-being and development': This category includes mentoring, coaching and leadership development and informal remote interactions as well as general and event-focused support to promote well-being among APs

Just under half of all retrieved work applied to achieving clinical outputs. Within this category, a substantial minority (41 papers; 18.2% of all included articles) were specifically related to intraoperative handover and its potential influence on clinical outcome. The remaining work was split approximately equally between papers on formal professional activity (most relating to teaching and training) and papers related to personal well-being and development, primarily focused on mentoring and support. Emergency situations were explicitly addressed in 27 (12%) of all articles, with regard to patients presenting for emergency surgery, or emergencies arising perioperatively.

## 4. Discussion

The objective of this scoping review was to describe the available evidence regarding communication among APs to meet clinical and professional goals, in terms of the modalities, settings, contexts and purposes of inter-AP communication, as well as which providers are involved. The overall review question was: What evidence exists on how APs communicate with one another to meet clinical and professional goals?

The main findings of this review can be summarised as follows. (i) Investigations of communication among APs generally treat communication as either a variable influencing patient or provider well-being, or an outcome to be improved by innovative (largely technology-based) approaches; relatively rarely is the availability of communication with other APs evaluated as a resource. (ii) Around 50% of the work examining communication among APs relates to their clinical activity in theatre, with smaller proportions addressing formal professional activity and personal well-being. (iii) The low-resource setting is poorly represented in this body of work despite being the highest risk context in which anaesthesia is delivered.

The results of this scoping review demonstrate the considerable breadth of professional circumstances in which effective communication between APs occurs, is demonstrably necessary or is assumed: from immediate emergent help-seeking to the development of long-term professional associations with roles including advocacy, education and quality assurance. We identified that work on communication between APs tends to fall into two main categories, either treating communication as a variable which may affect a further outcome of interest, or as the outcome of interest in itself. A small minority of papers also treated communication as a resource, stressing issues of access to inter-provider communication and methods of improving this access. This is likely to be particularly relevant to anaesthetists of all cadres working in more remote settings and with fewer physical or infrastructural resources to manage their patients, though very little work has been done to evaluate the role of social or communication resource in this context.

We mark the relative underrepresentation of the low-resource setting in this body of literature, which is consistent with existing knowledge of the imbalance in research outputs between high-income and low/middle-income countries [35, 36]. Such underrepresentation not only deprives the global community of context and understanding of the true global picture but also is likely to miss out the communities most at risk from inadequate inter-provider communication: patients requiring anaesthesia in remote and rural locations, and the APs themselves who are more likely to be isolated professionally and whose access to the social resource often assumed in high-income countries' professional contexts is unknown.

Linked with this finding is the negligible representation of nonphysician, nonnurse providers such as clinical officers in the literature on AP communication. Although a minority group within the global anaesthesia workforce overall, in many countries (particularly in sub-Saharan Africa), this ‘midlevel' cadre of providers form the majority of the national workforce [2] and serve the majority of the country's population, particularly those in remote and rural, low-resource regions [37]. Very little work has been done to understand their professional environment and their access to communication with other providers, despite their key role in anaesthesia provision.

From this scoping exercise, we consider that there is an insufficient evidence base currently to recommend particular practices around communication between APs. However the centrality of communication to a wide range of domains and contexts is clear, suggesting that further study of how providers communicate most effectively in clinical and nonclinical elements of their professional practice would be valuable. Further work to identify and evaluate interventions with potential to improve communication practices outside anaesthesia might also usefully feed into this area of study.

### 4.1. Limitations

This review has limitations. Firstly, it explores the scope of literature which highlights communication as a primary topic: It therefore excludes papers related to all the contexts described above which do not foreground communication in their write-up. We also recognise that the search strategy based around the three concepts identified as highly relevant to communication (COPs, interprofessional practice/task-shifting/TS and networks) may have influenced the review findings. It is possible that identifying other themes relevant to communication and including these in the search strategy could have widened the scope of the review.

Secondly, only English and French-language articles were included, reflecting the capacity of the authors. We acknowledge that this may have excluded relevant articles from the review, though we also note that no language limits were applied to the initial search, and subsequent screening only identified 63 articles not in English or French from the search results (the language breakdown is available in Supporting file 5: Articles excluded on the basis of language).

Thirdly, we recognise the relevance and critical importance of communication between AP and patient to effective and high-quality care, which fell outside the formal scope of this review (though has been addressed elsewhere [38]). We have purposefully restricted our scope to mapping the literature particular to intraprofession communication, which has not been previously reviewed in this way. We suggest that for onward strategy development and evaluation of interventions, learning from wider communication contexts involving APs (including with patients), as well as in healthcare more generally, will be invaluable.

## 5. Conclusions

The objective of this scoping review was to describe the available literature relating to communication between APs for clinical and professional purposes. We found that communication is variously treated as a modifiable variable, a desirable outcome or (more rarely) as an optimisable resource, in a wide range of domains related to all aspects of anaesthesia practice. By demonstrating a spectrum of clinical and professional communication modalities and purposes within the anaesthesia workforce, our findings suggest that access to communication with other APs should be viewed as a significant resource for the safe practice of anaesthesia. More research in this area, particularly in low- and middle-income contexts and with NPAPs, could positively influence both workforce capacity and patient care.

## Figures and Tables

**Table 1:** Content inclusion criteria at screening.

Element	Included	Definition
Participants	Anaesthesia providers	Includes: • Healthcare workers (HCWs) who are independently responsible for providing anaesthesia, from any cadre, e.g., physician anaesthesiologists, nurse anaesthetists and clinical officer anaesthetists. • HCWs in formal anaesthetic training programmes at the time of the study Does not include: • HCWs who only assist another provider (i.e., are not independently responsible for patient anaesthesia, such as the UK's operating department practitioners) • Patients or public
Concept	Communication	Includes: • All modes of communication (phone, in-person, social media, online messaging, email, etc.) • Both informal and formal contexts. • Communication involving individuals and groups
Context	1. Communication occurring between anaesthesia providers (rather than between APs and non-APs) 2. Communication oriented towards clinical and/or professional goals	Clinical and/or professional goals include: • Training, teaching, learning, CPD • Supervision of any kind • ‘Extra pair of hands' presence request • Referral, advice • Mentoring, debriefing, career support

**Table 2:** The domains and purposes of communication studied (% of total included articles in brackets).

Operational category	Primary domain	Purposes included	Original research	Review	Description of process/technology	Other		Total
Achieving clinical output								111 (49.3%)
	Delivering anaesthesia care	Fostering nontechnical skills, teamworking; delivering tele-anaesthesia; handovers of care	50 (22.2%)	13 (5.7%)	3 (1.3%)	6 (2.7%)	72 (32%)	
	Making the most of the workforce	Accessing expertise (referrals, consultations); task sharing/shifting; coordinating clinical activity	17 (7.6%)	4 (1.8%)	1 (0.4%)	9 (4%)	31 (13.8%)	
	HR and information management	Working conditions, induction, information management systems and specific information sharing processes	3 (1.3%)	1 (0.4%)	2 (0.9%)	2 (0.9%)	8 (3.6%)	

Formal professional activity								55 (24.4%)
	Teaching and training the workforce	Learning in clinical environments; distance learning; broadcast learning (podcasts, websites); technical skills teaching	11 (4.9%)	8 (3.6%)	2 (0.9%)	2 (0.9%)	23 (10.2%)	
	Supervision	Provision of supervision; assessing quality of supervision	14 (6.2%)	0	0	0	14 (6.2%)	
	Evaluation	Multisource feedback; feedback in training; logbook reviews	8 (3.6%)	1 (0.4%)	0	0	9 (4%)	
	Professional network activities	Conference promotion, membership; fostering collaborations; standards and guidelines	0	0	4 (1.8%)	5 (2.2%)	9 (4%)	

Personal well-being and development								59 (26.2%)
	Mentoring	Mentoring juniors/trainees/newly qualified practitioners; leadership development and coaching; mentoring in academic activity	13 (5.8%)	3 (1.3%)	5 (2.2%)	3 (1.3%)	24 (10.7%)	
	Support	Peer support, well-being, mitigating burnout, solidarity; post-event support and debriefing	14 (6.2%)	4 (1.8%)	1 (0.4%)	0	19 (8.4%)	
	Remote informal activities	Social media use; other digital interactions	7 (3.1%)	4 (1.8%)	2 (0.9%)	3 (1.3%)	16 (7.1%)	

## Data Availability

The data that support the findings of this review are available on reasonable request from the corresponding author (H.E.).

## Supporting Information

Additional files are available related to this review:

Supporting Information 1 Supporting file 1: PRISMA-ScR checklist.

Supporting Information 2 Supporting file 2: Example search strategy.

Supporting Information 3 Supporting file 3: Data charting tool.

Supporting Information 4 Supporting file 4: Citation list of included sources.

Supporting Information 5 Supporting file 5: Articles excluded on the basis of language.

Supporting Information 6 Supporting file 6: Visual abstract.
