# Supplementary material for: Communication Between Anaesthesia Providers for Clinical and Professional Purposes: A Scoping Review
Source: Anesthesiol Res Pract. 2025 Mar 6;2025:3598234. doi: 10.1155/anrp/3598234 (PMC11991797; doi:10.1155/anrp/3598234)
Supplement: Supporting Information 2 — Supporting file 2: Example search strategy. [file 3598234.f2.docx]

**Supplementary file 2: Example search strategy**

Database: Medline (Ovid MEDLINE® Epub Ahead of Print, In-Process & Other Non-Indexed Citations, Ovid MEDLINE® Daily and Ovid MEDLINE®) 1946 to present

Search Strategy: (30/09/2022)

--------------------------------------------------------------------------------

1 exp Anesthetists/ (4955)

2 anaesthetist*.tw. (6840)

3 anesthetist*.tw. (4565)

4 anaesthesiologist*.tw. (3679)

5 anesthesiologist*.tw. (25571)

6 (anaesthe* adj3 provider*).tw. (155)

7 (anesthe* adj3 provider*).tw. (1445)

8 (anaesthe* adj3 nurs*).tw. (770)

9 (anesthe* adj3 nurs*).tw. (2874)

10 (anaesthe* adj3 assistant*).tw. (103)

11 (anesthe* adj3 assistant*).tw. (161)

12 Postanesthesia Nursing/ (1087)

13 (perianesthe* adj3 nurs*).tw. (267)

14 (perianaesthe* adj3 nurs*).tw. (3)

15 (postanaesthe* adj3 nurs*).tw. (13)

16 (postanesthe* adj3 nurs*).tw. (123)

17 (peri anesthe* adj3 nurs*).tw. (7)

18 (peri anaesthe* adj3 nurs*).tw. (2)

19 (post anaesthe* adj3 nurs*).tw. (21)

20 (post anesthe* adj3 nurs*).tw. (26)

21 (recovery room* adj3 nurs*).tw. (106)

22 crna.tw. (3410)

23 krna.tw. (35)

24 1 or 2 or 3 or 4 or 5 or 6 or 7 or 8 or 9 or 10 or 11 or 12 or 13 or 14 or 15 or 16 or 17 or 18 or 19 or 20 or 21 or 22 or 23 (47693)

25 exp Communication/ (352502)

26 communicat*.tw. (357856)

27 exp Communications Media/ (376461)

28 phone*.tw. (45823)

29 telephon*.tw. (69851)

30 cellphone*.tw. (565)

31 mobile*.tw. (119823)

32 text*.tw. (194591)

33 social media.tw. (22517)

34 twitter*.tw. (5536)

35 tweet*.tw. (4432)

36 email*.tw. (12068)

37 e mail*.tw. (9587)

38 electronic mail.tw. (749)

39 internet.tw. (65207)

40 intranet*.tw. (950)

41 web.tw. (162032)

42 telecommunication*.tw. (5031)

43 teleconferenc*.tw. (1426)

44 tele conferenc*.tw. (26)

45 blog*.tw. (2258)

46 social medium.tw. (24)

47 youtube.tw. (3118)

48 you tube.tw. (22)

49 25 or 26 or 27 or 28 or 29 or 30 or 31 or 32 or 33 or 34 or 35 or 36 or 37 or 38 or 39 or 40 or 41 or 42 or 43 or 44 or 45 or 46 or 47 or 48 (1491006)

50 communities of practice.tw. (799)

51 community of practice.tw. (4025)

52 cops.tw. (809)

53 cop.tw. (7738)

54 Interprofessional Education/ (361)

55 exp Interprofessional Relations/ (72160)

56 interprofessional*.tw. (13364)

57 interdisciplinary.tw. (44107)

58 inter professional*.tw. (1716)

59 inter disciplinary.tw. (837)

60 multidisciplinary.tw. (107507)

61 multi disciplinary.tw. (8301)

62 exp Patient Care Team/ (72335)

63 teamwork*.tw. (12145)

64 Cooperative Behavior/ (45750)

65 cooperat*.tw. (156268)

66 co operat*.tw. (11845)

67 intersectoral collaboration/ (2567)

68 collaborat*.tw. (183192)

69 Mentors/ (12615)

70 Mentoring/ (3469)

71 mentor*.tw. (20201)

72 coordinat*.tw. (306881)

73 co ordinat*.tw. (11897)

74 task shar*.tw. (562)

75 task shift*.tw. (1274)

76 taskshar*.tw. (1)

77 taskshift*.tw. (3)

78 social network analysis/ (220)

79 network*.tw. (632691)

80 psychosocial support systems/ (925)

81 support*.tw. (1835092)

82 Help-Seeking Behavior/ (1129)

83 help-seeking.tw. (7377)

84 helpseeking.tw. (47)

85 (help adj3 seek*).tw. (13624)

86 Counseling/ (38979)

87 peer*.tw. (118202)

88 86 and 87 (1262)

89 (peer* adj3 counsel*).tw. (915)

90 50 or 51 or 52 or 53 or 54 or 55 or 56 or 57 or 58 or 59 or 60 or 61 or 62 or 63 or 64 or 65 or 66 or 67 or 68 or 69 or 70 or 71 or 72 or 73 or 74 or 75 or 76 or 77 or 78 or 79 or 80 or 81 or 82 or 83 or 84 or 85 or 88 or 89 (3154309)

91 24 and 49 and 90 (1440)

***************************
