# Supplementary material for: Communication Between Anaesthesia Providers for Clinical and Professional Purposes: A Scoping Review
Source: Anesthesiol Res Pract. 2025 Mar 6;2025:3598234. doi: 10.1155/anrp/3598234 (PMC11991797; doi:10.1155/anrp/3598234)
Supplement: Supporting Information 3 — Supporting file 3: Data charting tool. [file 3598234.f3.docx]

**Supplementary file 3: Data charting tool**

| title | year | journal | vol | issue | pages | authors | TYPE OF ANAESTHESIA PROVIDERS DISCUSSED | TYPE OF PAPER | COUNTRY_OR_IES INCLUDED | HIC OR LMIC |
| --- | --- | --- | --- | --- | --- | --- | --- | --- | --- | --- |
|  |  |  |  |  |  |  |  |  |  |  |
|  |  |  |  |  |  |  |  |  |  |  |

| MECHANISM OF COMMUNICATION | SITUATION OF COMMUNICATION | PURPOSE OF COMMUNICATION | PRIMARY _OR_ MAIN OUTCOME | OTHER OUTCOMES | PLACE OF COMMUNICATION IN STUDY |
| --- | --- | --- | --- | --- | --- |
|  |  |  |  |  |  |
|  |  |  |  |  |  |

| FACILITATORS OF COMMUNICATION (POSSIBLE OR ACTUAL) | BARRIERS TO COMMUNICATION (POSSIBLE_OR_ACTUAL) | COMMUNITY OF PRACTICE MENTIONED EXPLICITLY? | INTERPROFESSIONAL COMMUNICATION OR TASK SHARING MENTIONED EXPLICITLY? | NETWORK_OR_S (HUMAN, NOT TECHNOLOGICAL) MENTIONED EXPLICITLY? | OTHER THEORY _OR_ CONCEPTUAL FRAMEWORK (IF EXPLICIT) |
| --- | --- | --- | --- | --- | --- |
|  |  |  |  |  |  |
|  |  |  |  |  |  |
