# Supplementary material for: Communication Between Anaesthesia Providers for Clinical and Professional Purposes: A Scoping Review
Source: Anesthesiol Res Pract. 2025 Mar 6;2025:3598234. doi: 10.1155/anrp/3598234 (PMC11991797; doi:10.1155/anrp/3598234)
Supplement: Supporting Information 4 — Supporting file 4: Citation list of included sources. [file 3598234.f4.docx]

**Supplementary file 4: Citation list of included sources**

| Title | | Year* | Journal* | Vol* | Pages* | Authors |
| --- | --- | --- | --- | --- | --- | --- |
| Beyond the Surgical Safety Checklist: Using Intraoperative Handoff to Facilitate Team Situation Awareness in the OR | | 2023 | Annals of Surgery | 278 | e1142-e1147 | A. Ramjaun; M. Hammond Mobilio; N. Wright; M. Masella; A. Snyman; C. Serrick; C. A. Moulton |
| Computer-based anesthesiology paging system | | 2003 | Anesthesia & Analgesia | 97 | 196-204, table of contents | Abenstein, J. P. and Allan, J. A. and Ferguson, J. A. and Deick, S. D. and Rose, S. H. and Narr, B. J. |
| An evidence synthesis on perioperative handoffs: A call for balanced sociotechnical solutions. International Journal of Medical Informatics. 2023 Mar 8:105038. | |  |  |  |  | Abraham J, Duffy C, Kandasamy M, France D, Greilich P. |
| Systematic review of intraoperative anesthesia handoffs and handoff tools. Anesth Analg. 2021;132:1563–1575. | |  |  |  |  | Abraham J, Pfeifer E, Doering M, Avidan MS, Kannampallil T. |
| An electronic checklist improves transfer and retention of critical information at intraoperative handoff of care | | 2015 | Anesthesia & Analgesia | 120 | 96-104 | Agarwala, A. V. and Firth, P. G. and Albrecht, M. A. and Warren, L. and Musch, G. |
| Mentoring in Medical Education | | 2022 | Update in Anaesthesia | 36 | 47-53 | Akrimi, Sonia and Bould, M. Dylan |
| Pro-con debate: consideration of race, ethnicity, and gender is detrimental to successful mentorship | | 2023 | Anesthesia & Analgesia | 137 | 747-753 | Alicia B Barclay; Kenneth Moran; Dmitry Tumin; Kimberley R Nichols |
| A qualitative study exploring mentorship in anesthesiology: perspectives from both sides of the relationship. Can J Anesth. 2016; 63: 851-861. | |  |  |  |  | Alisic S, Boet S, Sutherland S, Bould MD. |
| Temporary Intraoperative Handoff Report Among Nurse Anesthetists: Utilization of Standardized Handoff Tools and Barriers to Implementation. AANA journal. 2023 Apr 1;91(2):130-6. | |  |  |  |  | Allen R, Nemec C, O'Guin C, Wright S, Dalley CB. |
| Anesthetic care of the trauma patient: development of a Web-based resource | | 2007 | AANA Journal | 75 | 49-56 | Anderson, N. K. and Jones, A. D. and Martin, E. E. and Thies, R. F. and Baxter, D. and Marienau, M. E. and Elliott, B. A. |
| Social Media in Nurse Anesthesia: A Model of a Reproducible Educational Podcast | | 2017 | AANA Journal | 85 | 01_OR_10_OR_2016 | Andrejco, K. and Lowrance, J. and Morgan, B. and Padgett, C. and Collins, S. |
| Mentoring Team Projects for the Doctor of Nursing Practice: Considerations for Nurse Anesthesia Faculty | | 2021 | AANA Journal | 89 | 435-442 | Aroke, E. N. and Wilbanks, B. A. and Hicks, T. and Thurston, K. L. and McMullan, S. P. |
| Toward a blueprint for perioperative handoffs and handoff tools. Anesthesia & Analgesia. 2021 Jun 1;132(6):1559-62. | |  |  |  |  | Arriaga AF, Chen YY, Kim JJ, Bader AM. |
| Failure to Debrief after Critical Events in Anesthesia Is Associated with Failures in Communication during the Event | | 2019 | Anesthesiology | 130 | 1039-1048 | Arriaga, A. F. and Sweeney, R. E. and Clapp, J. T. and Muralidharan, M. and Burson, R. C., 2nd and Gordon, E. K. B. and Falk, S. A. and Baranov, D. Y. and Fleisher, L. A. |
| Bridging the human resource gap in surgical and anesthesia care in low-resource countries: a review of the task sharing literature. Human resources for health. 2017 Dec;15(1):1-1. | |  |  |  |  | Ashengo T, Skeels A, Hurwitz EJ, Thuo E, Sanghvi H. |
| Health-promoting collaboration in anesthesia nursing: a qualitative study of nurse anesthetists in Norway | | 2012 | AANA Journal | 80 | S74-80 | Averlid, G. and Axelsson, S. B. |
| The operating room as a learning arena: Nurse anaesthetist and student nurse anaesthetist perceptions | | 2020 | Journal of Clinical Nursing | 29 | 1673-1683 | Averlid, G. and Hoglund, J. S. |
| Prevalence of anaesthesia information management systems in university-affiliated hospitals in Europe | | 2010 | European Journal of Anaesthesiology | 27 | 202-8 | Balust, J. and Egger Halbeis, C. B. and Macario, A. |
| Speak up! Barriers to challenging erroneous decisions of seniors in anaesthesia | | 2016 | Anaesthesia | 71 | 1332-1340 | Beament, T. and Mercer, S. J. |
| Pediatric Anesthesiology Fellows’ perception of quality of attending supervision and medical errors. Anesthesia & Analgesia. 2018 Feb 1;126(2):639-43. | |  |  |  |  | Benzon HA, Hajduk J, De Oliveira G, Suresh S, Nizamuddin SL, McCarthy R, Jagannathan N. |
| Teleconsultation for anaesthesiologists-Update and limitations. Current Medicine Research and Practice. 2022 Sep 1;12(5):238. | |  |  |  |  | Bhalotra AR. |
| Hierarchy in critical airway management | | 2016 |  | 71 | 111-112 | Bidwai, A. and Beament, T. and Mercer, S. |
| Handoff checklists improve the reliability of patient handoffs in the operating room and postanesthesia care unit. Paediatr Anaesth 2013; 23:647–654. | |  |  |  |  | Boat AC, Spaeth JP. |
| Association of intraoperative anaesthesia handovers with patient morbidity and mortality: A systematic review and meta-analysis. Br J Anaesth. 2020; 125: 605-613 | |  |  |  |  | Boet S Djokhdem H Leir SA et al. |
| Assessing the impact of surgical and anaesthetic task-shifting globally | |  |  |  |  | Bognini et al |
| Residents' reluctance to challenge negative hierarchy in the operating room: a qualitative study | | 2015 | Canadian Journal of Anaesthesia | 62 | 576-86 | Bould, M. D. and Sutherl and , S. and Sydor, D. T. and Naik, V. and Friedman, Z. |
| Certified Registered Nurse Anesthetist Working Conditions and Outcomes: A Review of the Literature | | 2017 | AANA Journal | 85 | 261-269 | Boyd, D. and Poghosyan, L. |
| Dear Doctor': a randomised controlled trial of a text message intervention to reduce burnout in trainee anaesthetists | | 2022 | Anaesthesia | 77 | 405-415 | Brazier, A. and Larson, E. and Xu, Y. and Judah, G. and Egan, M. and Burd, H. and Darzi, A. |
| To infinity and beyond: the past, present, and future of tele-anesthesia. Anesthesia & Analgesia. 2020 Feb 1;130(2):276-84. | |  |  |  |  | Bridges KH, McSwain JR, Wilson PR. |
| Teaching ultrasound-guided regional anesthesia remotely: a feasibility study | | 2016 | Acta Anaesthesiologica Scandinavica | 60 | 995-1002 | Burckett-St Laurent, D. A. and Cunningham, M. S. and Abbas, S. and Chan, V. W. and Okrainec, A. and Niazi, A. U. |
| Hold the Phone! Perioperative Personal Electronic Devices | | 2022 | Current Anesthesiology Reports | 12(4) | 476-483 | C. Moshe; C. R. Roscher; S. Porter |
| An Intraoperative Telemedicine Program to Improve Perioperative Quality Measures: The ACTFAST-3 Randomized Clinical Trial | | 2023 | JAMA Network Open | 6(9) | E2332517 | C. R. King; S. Gregory; B. A. Fritz; T. P. Budelier; A. Ben Abdallah; A. Kronzer; D. L. Helsten; B. Torres; S. McKinnon; S. Goswami; D. Mehta; O. Higo; P. Kerby; B. Henrichs; T. S. Wildes; M. C. Politi; J. Abraham; M. S. Avidan; T. Kannampallil |
| Perceptions, attitudes, and beliefs of staff anaesthetists related to multi-source feedback used for their performance appraisal | | 2011 | BJA: The British Journal of Anaesthesia | 107 | 372-377 | Castanelli, D. and Kitto, S. and Castanelli, D. and Kitto, S. |
| Telemedicine and anaesthesia. Indian journal of anaesthesia. 2010 May;54(3):199. | |  |  |  |  | Chatrath V, Attri JP, Chatrath R. |
| Intraoperative patient information handover between anesthesia providers. J Biomed Res 2014; 28:383 – 387. | |  |  |  |  | Choromanski D, Frederick J, McKelvey GM, et al. |
| Information technology and its role in anaesthesia training and continuing medical education. Best Pract Res Clin Anaesthesiol 2012; 26: 33-53 | |  |  |  |  | Chu LF, Erlendson MJ, Sun JS, Clemenson AM, Martin P, Eng RL. |
| Resident Physicians Improve Nontechnical Skills When on Operating Room Management and Leadership Rotation | | 2017 | Anesthesia & Analgesia | 124 | 300-307 | Cole, D. C. and Giordano, C. R. and Vasilopoulos, T. and Fahy, B. G. |
| Remote anesthetic monitoring using satellite telecommunications and the internet. Anesth Analg 2006; 102:1463 – 1467 | |  |  |  |  | Cone SW, Gehr L, Hummel R, Merrell RC. |
| Enhancing anaesthesiology training in Europe with a trainees' network | | 2017 | Trends in Anaesthesia and Critical Care | 14 | 30-32 | D. Sobreira Fernandes, and Hansen, D. A. |
| Practice of debriefing of critical events: a survey-based cross-sectional study of Portuguese anesthesiologists | | 2023 | Porto Biomedical Journal | 8 | e215 | D. Teles; M. Silva; J. Berger-Estilita; H. Pereira |
| Continuous monitoring and feedback of quality of recovery indicators for anaesthetists: a qualitative investigation of reported effects on professional behaviour | | 2017 | BJA: The British Journal of Anaesthesia | 119 | 115-124 | D'Lima, D. and Arnold, G. and Brett, S. J. and Bottle, A. and Smith, A. and Benn, J. |
| Improving the training process of anesthesiology residents through the mentorship-based approach. Anesthesiology and Pain Medicine. 2019 Feb;9(1). | |  |  |  |  | Dabbagh A, Massoudi N, Vosoghian M, Mottaghi K, Mirkheshti A, Tajbakhsh A, Sezari P, Moshari M, Tabashi S. |
| Mitigating burnout and enhancing wellness in anesthesiologists: Individual interventions, wellness programs, and peer support | | 2021 | International Anesthesiology Clinics | 59 | 73-80 | Dalton, A. |
| Intraoperative Handoff During Cardiac Surgery: A Fumble?. Journal of Cardiothoracic and Vascular Anesthesia. 2022 Aug 1;36(8):2851-3. | |  |  |  |  | Daubenspeck DK, Chaney MA. |
| An instrument designed for faculty supervision evaluation by anesthesia residents and its psychometric properties. Anesth Analg 2008;107:1316–22 | |  |  |  |  | de Oliveira Filho GR, Dal Mago AJ, Garcia JH, Goldschmidt R. |
| The association between frequency of selfreported medical errors and anesthesia trainee supervision: a survey of United States anesthesiology residents-in-training. Anesth Analg 2013;116:892–97 | |  |  |  |  | De Oliveira GS Jr, Rahmani R, Fitzgerald PC, Chang R, McCarthy RJ. |
| Reliability and validity of assessing subspecialty level of faculty anesthesiologists' supervision of anesthesiology residents | | 2015 | Anesthesia & Analgesia | 120 | 209-213 | De Oliveira, G. S., Jr. and Dexter, F. and Bialek, J. M. and McCarthy, R. J. |
| Overall anesthesia department quality of clinical supervision of trainees over a year evaluated using mixed effects models. Journal of clinical anesthesia. 2023 Aug 1;87:111114. | |  |  |  |  | Dexter F, Hindman BJ, Epstein RH. |
| Operating room anesthesia subspecialization is not associated with significantly greater quality of supervision of anesthesia residents and nurse anesthetists. Anesthesia & Analgesia. 2017 Apr 1;124(4):1253-60. | |  |  |  |  | Dexter F, Ledolter J, Epstein RH, Hindman BJ. |
| Bernoulli cumulative sum (CUSUM) control charts for monitoring of anesthesiologists' performance in supervising anesthesia residents and nurse anesthetists. Anesth Analg 2014;119:679–85. | |  |  |  |  | Dexter F, Ledolter J, Hindman BJ. |
| Influence of provider type (nurse anesthetist or resident physician), staff assignments, and other covariates on daily evaluations of anesthesiologists' quality of supervision. Anesth Analg 2014;119:670–8. | |  |  |  |  | Dexter F, Ledolter J, Smith TC, Griffiths D, Hindman BJ. |
| Anesthesiology residents’ and nurse anesthetists’ perceptions of effective clinical faculty supervision by anesthesiologists. Anesth Analg 2013;116:1352–5 | |  |  |  |  | Dexter F, Logvinov II, Brull SJ. |
| Work habits are valid components of evaluations of anesthesia residents based on faculty anesthesiologists’ daily written comments about residents. Anesth Analg. 2016;122:1625–1633 | |  |  |  |  | Dexter F, Masursky D, Szeluga D, Hindman BJ. |
| Written comments made by anesthesia residents when providing below average scores for the supervision provided by the faculty anesthesiologist. Anesth Analg 2016;122:1999–2005. | |  |  |  |  | Dexter F, Szeluga D, Masursky D, Hindman BJ. |
| Quality of Supervision as an Independent Contributor to an Anesthesiologist's Individual Clinical Value | | 2015 | Anesthesia & Analgesia | 121 | 507-13 | Dexter, F. and Hindman, B. J. |
| Nurse anesthetists' preferences for anesthesiologists' participation in patient care at a large teaching hospital | | 2019 | Journal of Clinical Anesthesia | 57 | 131-138 | Dexter, F. and Ledolter, J. and Wong, C. A. and O'Brien, M. K. and Hindman, B. J. |
| An audit of the efficacy of a structured handover tool in obstetric anaesthesia. Int J Obstet Anesth. 2014 May;23(2):151-156. doi:10.1016_OR_j.ijoa.2013.12.002 | |  |  |  |  | Dharmadasa A, Bailes I, Gough K, Ebrahimi N, Robinson PN, Lucas DN. |
| Communication failures contributing to patient injury in anaesthesia malpractice claims☆. British Journal of Anaesthesia. 2021 Sep 1;127(3):470-8. | |  |  |  |  | Douglas RN, Stephens LS, Posner KL, Davies JM, Mincer SL, Burden AR, Domino KB. |
| Not if, but how? Using technology and task-sharing to strengthen the global anesthesia workforce. Anesthesia & Analgesia. 2017 Oct 1;125(4):1095-7. | |  |  |  |  | Drum ET, Bould MD. |
| Remote obstetric anesthesia: leveraging telemedicine to improve fetal and maternal outcomes. Telemedicine and e-Health. 2020 Aug 1;26(8):967-72. | |  |  |  |  | Duarte SS, Nguyen TA, Koch C, Williams K, Murphy JD. |
| Social media and anaesthesia journals. Br J Anaesth 2015;115:940-1. | |  |  |  |  | Duffy CC, Bass GA, Linton KN, Honan DM. |
| Does Moral Distress Differ Between California Certified Registered Nurse Anesthetists in Independent Versus Medically Supervised Practice: An Exploratory Study | | 2015 | AANA Journal | 83 | 203-9 | Dumouchel, M. and Boytim, M. and Gorman, N. and Weismuller, P. |
| Human resources in anesthesia: the road to 2030. Anesthesia & Analgesia. 2017 Sep 1;125(3):734-6. | |  |  |  |  | Enright A, Newton M. |
| Mentorship in anesthesia: a survey of perspectives among Canadian anesthesia residents. Can J Anesth. 2017; 64: 402-410. | |  |  |  |  | Ergun S, Busse J, Wong A. |
| Evaluation of Open Access Websites for Anesthesia Education | | 2022 | Anesthesia & Analgesia | 18 | 18 | Evans, F. M. and Krotinger, A. A. and Lilaonitkul, M. and Khaled, H. F. and Pereira, G. A. and Staffa, S. J. and Wolbrink, T. A. |
| A survey of mentoring patterns, practices, and attitudes of active medical school-affiliated anaesthesiologists in the USA. British Journal of Anaesthesia. 2023 Feb 1;130(2):e191-3. | |  |  |  |  | Faloye AO, Bechtel AJ, Boorman DW, Methangkool E. |
| Formation of an Anesthesiology Residency Mentoring Program Using a Mentee-Driven Approach. A&A Practice. 2021 Jun 1;15(6):e01474. | |  |  |  |  | Faloye AO, Williamson J, Arora S, Nafiu OO, Peterson-Layne C. |
| Assessment of an Anesthesiology Academic Department Mentorship Program. The Ochsner Journal. 2012; 12: 373–378 | |  |  |  |  | Farag E, Abd-Elsayed A, Mascha E, O’Hara J. |
| Exploring Strategies to Increase and Sustain Membership in the American Association of Nurse Anesthetists | | 2016 | AANA Journal | 84 | 396-403 | Farina, Cynthia Ann |
| Telemedicine consultation and monitoring for pediatric liver transplant. Anesth Analg 2009;108:1212–4 | |  |  |  |  | Fiadjoe J, Gurnaney H, Muralidhar K, et al. |
| Telemedicine, Anesthesia, and Global Health. Current Anesthesiology Reports. 2017 Mar;7:8-14. | |  |  |  |  | Firth PG, Joseph SE. |
| Evaluation of intraoperative hand-off frequency, duration, and context: a mixed methods analysis. Journal of Surgical Research. 2020 Dec 1;256:124-30. | |  |  |  |  | Frasier LL, Quamme SR, Wiegmann D, Greenberg CC. |
| Familiarity and Communication in the Operating Room | | 2019 | Journal of Surgical Research | 235 | 395-403 | Frasier, L. L. and Pavuluri Quamme, S. R. and Ma, Y. and Wiegmann, D. and Leverson, G. and DuGoff, E. H. and Greenberg, C. C. |
| Challenging authority during an emergency—the effect of a teaching intervention. Critical care medicine. 2017 Aug 1;45(8):e814-20. | |  |  |  |  | Friedman Z, Perelman V, McLuckie D, Andrews M, Noble LM, Malavade A, Bould MD. |
| Power and conflict: the effect of a superior's interpersonal behaviour on trainees' ability to challenge authority during a simulated airway emergency | | 2015 | Anaesthesia | 70 | 1119-29 | Friedman, Z. and Hayter, M. A. and Everett, T. C. and Matava, C. T. and Noble, L. M. and Bould, M. D. |
| Implementation of a SAFE OB Handover for CRNAs | | 2022 | AANA Journal | 90 | 17-24 | Gabot, Mark H. |
| Twitter hashtags for anesthesiologists: building global communities. A&A Practice. 2019 Jan 15;12(2):59-62. | |  |  |  |  | Gai N, Matava C. |
| Telemedicine in anesthesia: an update | | 2011 | Current Opinion in Anaesthesiology | 24 | 459-62 | Galvez, J. A. and Rehman, M. A. |
| Social embeddedness of firefighters, paramedics, specialized nurses, police officers, and military personnel: Systematic review in relation to the risk of traumatization. Frontiers in Psychiatry. 2020 Dec 21;11:496663. | |  |  |  |  | Geuzinge R, Visse M, Duyndam J, Vermetten E. |
| Mentorship in anesthesia: A perspective survey among anesthesia residents in Riyadh, Saudi Arabia. Saudi Journal of Anaesthesia. 2021 Apr;15(2):144. | |  |  |  |  | Ghali KN, AlSubaie AT, Nawab AA. |
| The effectiveness of an anesthesia handoff tool: An electronic health record application to enhance patient safety. Anesthesia eJournal. 2016 May 13;4(1). | |  |  |  |  | Gillikin KE, Apatov N. |
| A survey of residency program directors in anesthesiology regarding mentorship of residents | | 2016 | Journal of Clinical Anesthesia | 33 | 254-265 | Gonzalez, L. S. and Donnelly, M. J. |
| The role of feedback in ameliorating burnout | | 2018 | Current Opinion in Anesthesiology | 31 | 361-365 | Gordon, Emily K. and Baranov, Dimitry Y. and Fleisher, Lee A. |
| Implementation of an Innovative Residency Leadership Development Curriculum | | 2019 | A&A Practice | 12 | 252-255 | Grantham, A. and Hauck, J. and Stafford-Smith, M. and Mathew, J. P. and Thompson, A. |
| Come together'. How the AAGBI reaches consensus over its guidelines | | 2016 | Anaesthesia | 71 | 261-264 | Griffiths, R. and Shinde, S. and Clyburn, P. |
| Characteristics of nurse anesthetists working with and without anesthesiologists | | 1987 | Medical Care | 25 | 1129-38 | Grundy, B. L. and Medsger, A. and Silverman, M. and Ricci, E. and Bottoms, C. and Gunter, M. J. and Callahan, C. |
| Distraction in the operating room: a narrative review of environmental and self-initiated distractions and their effect on anesthesia providers. Journal of Clinical Anesthesia. 2021 Feb 1;68:110110. | |  |  |  |  | Gui JL, Nemergut EC, Forkin KT. |
| Contemporary social network sites: Relevance in anesthesiology teaching, training, and research. J Anaesthesiol Clin Pharmacol 2016;32:382-5. | |  |  |  |  | Haldar R, Kaushal A, Samanta S, Ambesh P, Srivastava S, Singh PK, et al. |
| Association of anesthesiologist handovers with short-term outcomes for patients undergoing cardiac surgery. Anesthesia & Analgesia. 2020 Nov 13;131(6):1883-9. | |  |  |  |  | Hannan EL, Samadashvili Z, Sundt TM, Girardi L, Chikwe J, Wechsler A, Adams DH, Smith CR, Gold JP, Lahey SJ, Jordan D. |
| In the Aftermath: attitudes of Anesthesiologists to Supportive Strategies after an Unexpected Intraoperative Patient Death | | 2016 | Anesthesia and analgesia | 122 | 1614‚Äê1624 | Heard, G. C. and Thomas, R. D. and erson, P. M. |
| Educational support for specialist international medical graduates in anaesthesia | | 2013 | Medical Journal of Australia | 199 | 272-4 | Higgins, N. S. and Taraporewalla, K. and Edirippulige, S. and Ware, R. S. and Steyn, M. and Watson, M. O. |
| Anesthesia residents’ global (departmental) evaluation of faculty anesthesiologists’ supervision can be less than their average evaluations of individual anesthesiologists. Anesthesia & Analgesia. 2015 Jan 1;120(1):204-8. | |  |  |  |  | Hindman BJ, Dexter F, Smith TC. |
| Handover of responsibility for the anaesthetised patient—opinion and practice. Anaesthesia 2004;59:658–63 | |  |  |  |  | Horn J, Bell MD, Moss E. |
| Adverse outcomes associated with intraoperative anesthesia handovers: a systematic review and meta-analysis. Journal of PeriAnesthesia Nursing. 2020 Oct 1;35(5):525-32. | |  |  |  |  | Hu J, Yang Y, Li X, Yu L, Zhou Y, Fallacaro MD, Wright S. |
| Building an International Partnership to Develop Advanced Practice Nurses in Anesthesia Settings: Using a Theory-Driven Approach | | 2019 | Journal of Transcultural Nursing | 30 | 521-529 | Hu, Jiale and Yang, Yan and Fallacaro, Michael D. and W and s, Brenda and Wright, Suzanne and Zhou, Yiyan and Ruan, Hong |
| Impact of anesthetic handover on mortality and morbidity in cardiac surgery: a cohort study. J Cardiothorac Vasc Anesth 2015; 29:11–16. | |  |  |  |  | Hudson CCC, McDonald B, Hudson JKC, et al. |
| Task sharing in global anesthesia and surgery: workforce concerns. Current Anesthesiology Reports. 2021 Mar;11:59-63. | |  |  |  |  | Igaga EN, Sendagire C, Ayebale ET. |
| Certified Registered Nurse Anaesthetists' and Critical Care Registered Nurses' perception of knowledge/power in teamwork with Anaesthesiologists in Sweden: a mixed-method study | | 2024 | BMC Nursing | 23 | 7 | J. Wising; M. Strom; J. Hallgren; K. Rambaree |
| Transcending intractable conflict in health care: an exploratory study of communication and conflict management among anesthesia providers | | 2003 | Journal of Health Communication | 8 | 563-81 | Jameson, J. K. |
| Evaluating safety of handoffs between anesthesia care providers. Ochsner J 2011;11:99–101 | |  |  |  |  | Jayaswal S, Berry L, Leopold R, Hart SR, Scuderi-Porter H, Digiovanni N, Phillips A. |
| Association between handover of anesthesia care and adverse postoperative outcomes among patients undergoing major surgery. Jama. 2018 Jan 9;319(2):143-53. | |  |  |  |  | Jones PM, Cherry RA, Allen BN, Jenkyn KM, Shariff SZ, Flier S, Vogt KN, Wijeysundera DN. |
| CRNA-physician collaboration in anesthesia | | 2009 | AANA Journal | 77 | 431-436 | Jones, T. S. and Fitzpatrick, J. J. |
| Training in intraoperative handover and display of a checklist improve communication during transfer of care: An interventional cohort study of anaesthesia residents and nurse anaesthetists | | 2017 | European Journal of Anaesthesiology | 34 | 471-476 | Jullia, M. and Tronet, A. and Fraumar, F. and Minville, V. and Fourcade, O. and Alacoque, X. and LeManach, Y. and Kurrek, M. M. |
| Association between paediatric intraoperative anaesthesia handover and adverse postoperative outcomes. BMJ Quality & Safety. 2021 Sep 1;30(9):755-63. | |  |  |  |  | Kannampallil T, Lew D, Pfeifer EE, Sharma A, Abraham J. |
| The Washington State nurse anesthetist workforce: a case study | | 2007 | AANA Journal | 75 | 37-42 | Kaplan, L. and Brown, M. and Andrilla, H. and Hart, L. G. |
| Social media and online communities of practice in anaesthesia education. Anaesthesia. 2019 Sep;74(9):1202-3. | |  |  |  |  | Kearsley R, MacNamara C. |
| Anaesthesiologist and social media: Walking the fine line | | 2018 | Indian Journal of Anaesthesia | 62 | 743-746 | Kiran, S. and Sethi, N. |
| Synchronous distance anesthesia education by internet videoconference between Uganda and the United States. J Clin Anesth. 2015;27(6):499–503. https:_OR__OR_ doi.org_OR_10.1016_OR_j.jclinane.2015.04.004 | |  |  |  |  | Kiwanuka JK, Ttendo SS, Eromo E, et al. |
| Knowledge, safety, and teamwork: a qualitative study on the experiences of anaesthesiologists and nurse anaesthetists working in the preanaesthesia assessment clinic. BMC anesthesiology. 2022 Oct 3;22(1):309. | |  |  |  |  | Kristoffersen EW, Opsal A, Tveit TO, Fossum M. |
| Strengthening the anesthesia workforce in low-and middle-income countries. Anesthesia & Analgesia. 2018 Apr 1;126(4):1291-7. | |  |  |  |  | Kudsk-Iversen S, Shamambo N, Bould MD. |
| The future of education in anesthesiology is social. International Anesthesiology Clinics. 2020 Oct 1;58(4):52-7. | |  |  |  |  | Kumar AH, Udani AD, Mariano ER. |
| What are we missing? The quality of intraoperative handover before and after introduction of a checklist. Canadian Journal of Anesthesia_OR_Journal canadien d'anesthésie. 2022 Jul;69(7):832-40. | |  |  |  |  | Lane S, Gross M, Arzola C, Malavade A, Szadkowski L, Huszti E, Friedman Z. |
| Are intraoperative anesthesia handovers associated with harm? Getting to the heart of the matter in cardiac surgery: the search for the hat-trick of quality, safety, and continuous improvement. Journal of cardiothoracic and vascular anesthesia. 2015 Feb 1;29(1):8-10. | |  |  |  |  | Lane-Fall M, Gutsche JT, Augoustides JG. |
| No matter the perspective, anesthesia handoffs are problematic. Anesthesia & Analgesia. 2016 Jan 1;122(1):7-9. | |  |  |  |  | Lane-Fall MB. |
| Enjoying work or burdened by it? How anaesthetists experience and handle difficulties at work: a qualitative study. British Journal of Anaesthesia. 2007 Oct 1;99(4):493-9. | |  |  |  |  | Larsson J, Rosenqvist U, Holmström I. |
| Being a young and inexperienced trainee anesthetist: a phenomenological study on tough working conditions | | 2006 | Acta Anaesthesiologica Scandinavica | 50 | 653-8 | Larsson, J. and Rosenqvist, U. and Holmstrom, I. |
| Navigating the challenges of performing anesthesia handoffs and conducting anesthesia handoff research. International Anesthesiology Clinics. 2020 Jan 1;58(1):32-7. | |  |  |  |  | Lazzara EH, Keebler JR, Simonson RJ, Agarwala A, Lane-Fall MB. |
| Analysis of variance of communication latencies in anesthesia: comparing means of multiple log-normal distributions | | 2011 | Anesthesia & Analgesia | 113 | 888-96 | Ledolter, J. and Dexter, F. and Epstein, R. H. |
| Electronic tool helps anaesthesia trainee handovers. The Clinical Teacher. 2019 Feb;16(1):58-63. | |  |  |  |  | Lee SC, Atkinson ME, Minard CG, O'Brien A. |
| Associations of form and function of speaking up in anaesthesia: a prospective observational study. British Journal of Anaesthesia. 2021 Dec 1;127(6):971-80. | |  |  |  |  | Lemke R, Burtscher MJ, Seelandt JC, Grande B, Kolbe M. |
| The need for a global perspective on task-sharing in anesthesia | | 2017 | Anesthesia & Analgesia | 125 | 1049-1052 | Lipnick, Michael S and Bulamba, Fred and Ttendo, Stephen and Gelb, Adrian W |
| Handover of anesthesia care is associated with an increased risk of delirium in elderly after major noncardiac surgery: results of a secondary analysis. Journal of anesthesia. 2019 Apr 19;33:295-303. | |  |  |  |  | Liu GY, Su X, Meng ZT, Cui F, Li HL, Zhu SN, Wang DX. |
| Safer obstetric anesthesia through education and mentorship:a model for knowledge translation in Rwanda. Canadian J Anaesthesia. 2014;61(11):1028–39 | |  |  |  |  | Livingston P, Evans F, Nsereko E, Nyirigira G, Ruhato P, Sargeant J,et al. |
| Non-technical skills of anaesthesia providers in Rwanda: an ethnography | | 2014 | The Pan African medical journal | 19 | 97 | Livingston, P. and Zolpys, L. and Mukwesi, C. and Twagirumugabe, T. and Whynot, S. and MacLeod, A. |
| Healthcare professionals' perception of their working environment and how to handle mental strain | | 2023 | Acta Anaesthesiologica Scandinavica | 67 | 979-986 | M. D. Madsen; P. Cedergreen; J. Nielsen; D. Ostergaard |
| Gender and Race/Ethnicity dynamics in anesthesiology mentorship: results of a European survey | | 2024 | BMC Anesthesiology | 24 | 311 | M. Gisselbaek; B. Marsh; L. Soriano; S. Jackman; L. Seidel; A. Albert; I. Matot; S. Coppens; S. Narouze; O. L. Barreto Chang; S. Saxena |
| The 'go-between' study: a simulation study comparing the 'Traffic Lights' and 'SBAR' tools as a means of communication between anaesthetic staff | | 2016 | Anaesthesia | 71 | 764-72 | MacDougall-Davis, S. R. and Kettley, L. and Cook, T. M. |
| Coordination patterns related to high clinical performance in a simulated anesthetic crisis | | 2009 | Anesthesia & Analgesia | 108 | 1606-15 | Manser, T. and Harrison, T. K. and Gaba, D. M. and Howard, S. K. |
| Grow and Advance through Intentional Networking (GAIN): a pilot program to foster connections within the Women’s Empowerment and Leadership Initiative (WELI) in the Society for Pediatric Anesthesia. Paediatr Anaesth. 2021. 2021;31:944-952 | |  |  |  |  | Margolis RD, Berenstain LK, Janosy N, et al. |
| The effect of two cognitive aid designs on team functioning during intra-operative anaphylaxis emergencies: a multi-centre simulation study | | 2016 | Anaesthesia | 71 | 389-404 | Marshall, S. D. and S and erson, P. and McIntosh, C. A. and Kolawole, H. |
| Educating Anesthesiologists During the Coronavirus Disease 2019 Pandemic and Beyond | | 2021 | Anesthesia & Analgesia | 132 | 585-593 | Martinelli, S. M. and Chen, F. and Isaak, R. S. and Huffmyer, J. L. and Neves, S. E. and Mitchell, J. D. |
| Videolaryngoscopes: From a vision problem to a communication problem | | 2017 | Anaesthesia, Pain and Intensive Care | 21 | 292-296 | Martinez-Hurtado, E. D. and Sanchez-Merchante, M. |
| Matava CT, Rosen D, Siu E, et al. |  |  |  |  |  |  |
| Non-technical skills progression during anesthesiology residency in Portugal: the impact of a National Pedagogical Plan | | 2020 | Medical Education Online | 25 | 1800980 | Matos, F. M. and Martins, M. R. and Martins, I. |
| Anesthesiology Point of Care project | | 2002 | Studies in Health Technology & Informatics | 85 | 287-9 | McDonald, J. S. and Noback, C. R. and Cheng, D. and Lee, T. K. and Nenov, V. |
| Increased use of Twitter at a medical conference: a report and a review of the educational opportunities | | 2012 | Journal of Medical Internet Research | 14 | e176 | McKendrick, D. R. and Cumming, G. P. and Lee, A. J. |
| Effect of intraoperative handovers of anesthesia care on mortality, readmission, or postoperative complications among adults: The HandiCAP Randomized Clinical Trial. JAMA. 2022 Jun 28;327(24):2403-12. | |  |  |  |  | Meersch M, Weiss R, Küllmar M, Bergmann L, Thompson A, Griep L, Kusmierz D, Buchholz A, Wolf A, Nowak H, Rahmel T. |
| Influence of transactive memory on perceived performance, job satisfaction and identification in anaesthesia teams | | 2008 | British Journal of Anaesthesia | 100 | 327-32 | Michinov, E. and Olivier-Chiron, E. and Rusch, E. and Chiron, B. |
| Improving faculty feedback to resident trainees during a simulated case: a randomized, controlled trial of an educational intervention. Anesthesiology. 2014;120:160–171. | |  |  |  |  | Minehart RD, Rudolph J, Pian-Smith MC, Raemer DB. |
| Faculty development in feedback provision. Int An&& esthesiol Clin 2016; 54:54 – 65 | |  |  |  |  | Mitchell JD, Jones SB. |
| FaceTime for teaching ultrasound-guided anesthetic procedures in remote place. J Clin Monit Comput 2014;28:211–5 | |  |  |  |  | Miyashita T, Iketani Y, Nagamine Y, Goto T. |
| A pilot study of tele-anaesthesia by virtual private network between an island hospital and a mainland hospital in Japan | | 2015 | Journal of Telemedicine & Telecare | 21 | 73-9 | Miyashita, T. and Mizuno, Y. and Sugawara, Y. and Nagamine, Y. and Koyama, Y. and Miyazaki, T. and Uchimoto, K. and Iketani, Y. and Tojo, K. and Goto, T. |
| The BUDDYS System: A Unique Peer Support Strategy Among Anaesthesiology Residents During the COVID-19 Pandemic. Turkish Journal of Anaesthesiology and Reanimation. 2022 Apr;50(Suppl 1):S62. | |  |  |  |  | Mohamed BA, Fowler WK, Thakkar M, Fahy BG. |
| Evaluation of a Managed Surgical Consultation Network in Malawi | | 2021 | World Journal of Surgery | 45 | 356-361 | Mwapasa, G. and Pittalis, C. and Clarke, M. and Bijlmakers, L. and Le, G. and Mk and awire, N. and Brugha, R. and Borgstein, E. and Gajewski, J. |
| Evaluating the Women's Empowerment and Leadership Initiative: Supporting mentorship, career satisfaction, and well-being among pediatric anesthesiologists | | 2023 | Paediatric Anaesthesia | 33 | 6-16 | N. Deutsch; S. D. Yanofsky; S. D. Markowitz; S. Tackett; L. K. Berenstain; L. I. Schwartz; R. Flick; E. Heitmiller; J. Fiadjoe; H. H. Lee; A. Honkanen; S. Malviya; J. K. Lee; J. M. Schwartz |
| Mentoring programs in academic anesthesiology: A case for PROFOUND mentoring for underrepresented minority faculty. Anesth Analg. 2019; 129(1): 316-320 | |  |  |  |  | Nafiu O and Haydar B. |
| Anaesthesiology trainers’ knowledge, attitudes and practices of feedback in a South African anaesthesiology department. Southern African Journal of Anaesthesia and Analgesia. 2021;27(4):180-5. | |  |  |  |  | Naicker K, Govender K, Singaram VS. |
| Advances in anesthesia education: increasing access and collaboration in medical education, from E-learning to telesimulation. Current Opinion in Anesthesiology. 2020 Dec 1;33(6):800-7. | |  |  |  |  | Nelsen BR, Chen YY, Lasic M, Bader AM, Arriaga AF. |
| Second victim support structures in anaesthesia: a cross-sectional survey in Belgian anaesthesiologists | | 2021 | International Journal for Quality in Health Care | 33 | 8 | Nijs, K. and Seys, D. and Coppens, S. and Van De Velde, M. and Vanhaecht, K. |
| Remote clinical support by telephone for rural district hospital medical officers in the Eastern Cape | | 2015 | South African Family Practice | 57 | 286-290 | Nqala, M. O. and Rout, C. C. and Aldous, C. M. |
| The Anaesthetic Friendship Society-supporting anaesthetic clinical officers in the southern districts of Malawi. Malawi Medical Journal. 2013 Sep;25(3):90. | |  |  |  |  | O'Regan M, Pearson V, Pollach G. |
| Lack of association between intraoperative handoff of care and postoperative complications: a retrospective observational study. BMC anesthesiology. 2019 Dec;19(1):1-8. | |  |  |  |  | O’Reilly-Shah VN, Melanson VG, Sullivan CL, Jabaley CS, Lynde GC. |
| Anaesthesia residents’ perception of the quality of patient handover: A multicentre, nonrandomised, high-fidelity simulation study. European Journal of Anaesthesiology\| EJA. 2022 May 1;39(5):477-80. | |  |  |  |  | Ouattara J, Fromentin M, Dinh AT, Baillard C, Fischler M, Gayat E, Le Guen M. |
| The Bullying in Anaesthesia Registrars Survey (BARS): Does a validated questionnaire improve our understanding of bullying in Australian and New Zealand anaesthesia trainees? | | 2023 | Anaesthesia and Intensive Care | 51(3) | 199-206 | P. B. J. Garnett; S. G. Douglas; R. H. Riley; L. J. Roberts |
| Professionalism and social networking: can patients, physicians, nurses, and supervisors all be 'friends?' | | 2012 | Health Care Manager | 31 | 285-94 | Peluchette, J. and Karl, K. and Coustasse, A. and Emmett, D. |
| Teaching residents the two-challenge rule: a simulation-based approach to improve education and patient safety | | 2009 | Simulation in Healthcare: The Journal of The Society for Medical Simulation | 4 | 84-91 | Pian-Smith, M. C. and Simon, R. and Minehart, R. D. and Podraza, M. and Rudolph, J. and Walzer, T. and Raemer, D. |
| Evaluation of Medical Confidentiality Breaches on Twitter Among Anesthesiology and Intensive Care Health Care Workers. Anesthesia & Analgesia. 2023 May 25:10-213. | |  |  |  |  | Pineau I, Pineau M, Selim J, Compère V, Besnier E, Zoé D, Popoff B, Clavier T. |
| A survey of mentor gender preferences amongst anesthesiology residents at the University of British Columbia. Canadian Journal of Anesthesia_OR_Journal canadien d'anesthésie. 2019 Mar 15;66:342-3. | |  |  |  |  | Plyley T, Cory J, Lorello GR, Flexman AM. |
| Near-peer mentorship compared with traditional mentorship in the University of British Columbia anesthesia residency program. Canadian Journal of Anesthesia_OR_Journal canadien d'anesthésie. 2020 Nov;67:1655-7. | |  |  |  |  | Plyley TJ, Cory JM, Flexman AM. |
| Cardiomyopathy in pregnancy and caesarean section: four case reports | | 2007 | International Journal of Obstetric Anesthesia | 16 | 68-73 | Pryn, A. and Bryden, F. and Reeve, W. and Young, S. and Patrick, A. and McGrady, E. M. |
| Improving Anesthesiologists' Ability to Speak Up in the Operating Room: A Randomized Controlled Experiment of a Simulation-Based Intervention and a Qualitative Analysis of Hurdles and Enablers | | 2016 | Academic Medicine | 91 | 530-9 | Raemer, D. B. and Kolbe, M. and Minehart, R. D. and Rudolph, J. W. and Pian-Smith, M. C. |
| Anesthesiologist fails to communicate with CRNA | | 1990 | Regan Report on Nursing Law | 30 | 02_OR_02_OR_2022 | Regan |
| The effects of a hands-free communication device system in a surgical suite | | 2011 | Journal of the American Medical Informatics Association | 18 | 70-2 | Richardson, J. E. and Shah-Hosseini, S. and Fiadjoe, J. E. and Ash, J. S. and Rehman, M. A. |
| Anesthesiology Patient Handoff Education Interventions: A Systematic Review. The Joint Commission Journal on Quality and Patient Safety. 2022 Dec 15. | |  |  |  |  | Riesenberg LA, Davis R, Heng A, do Rosario CV, O'Hagan EC, Lane-Fall M. |
| Norwegian standard for the safe practice of anaesthesia | | 2018 | Acta Anaesthesiologica Scandinavica | 62 | 411-417 | Ringvold, E. ‚ÄêM and Bekkevold, M. and Bruun, A. ‚ÄêM G. and B√∏rke, W. B. and Finjarn, T. J. and Haugen, A. S. and Isern, E. and Skjeflo, G. W. and Ulvik, A. and Ringvold, E. M. and Bruun, A. M. G. |
| Operating room relay strategy for turnover time improvement: a quality improvement project | | 2022 | BMJ Open Quality | 11 | 7 | Riveros Perez, E. and Kerko, R. and Lever, N. and White, A. and Kahf, S. and Avella-Molano, B. |
| Paging doctor, emergency?. Anesthesia & Analgesia. 2014 Jul 1;119(1):4-6. | |  |  |  |  | Rothman BS, Ehrenfeld JM. |
| Communication latencies of Apple push notification messages relevant for delivery of time-critical information to anesthesia providers | | 2013 | Anesthesia & Analgesia | 117 | 398-404 | Rothman, B. S. and Dexter, F. and Epstein, R. H. |
| The anesthesiology discussion group: Development of a new method of communication between anesthesiologists. Anesth Analg 1995; 81:163^166 | |  |  |  |  | Ruskin KJ, Kofke WA, Turndorf H. |
| Communication devices in the operating room | | 2006 | Current Opinion in Anaesthesiology | 19 | 655-9 | Ruskin, K. J. |
| Internet teleconferencing as a clinical tool for anesthesiologists | | 1998 | Journal of Clinical Monitoring & Computing | 14 | 183-9 | Ruskin, K. J. and Palmer, T. E. and Hagenouw, R. R. and Lack, A. and Dunnill, R. |
| Intraoperative transitions of anesthesia care and postoperative adverse outcomes. Anesthesiology 2014; 121:695 – 706. | |  |  |  |  | Saager L, Hesler BD, You J, et al. |
| A national survey of obstetric anaesthetic handovers. Anaesthesia 2006;61:376–80 | |  |  |  |  | Sabir N, Yentis SM, Holdcroft A. |
| Workplace Aggression: Assessment of Prevalence in the Field of Nurse Anesthesia | | 2011 | AANA Journal | 79 | S51-S57 | Sakellaropoulos, Amelia and Pires, Janine and Estes, Donna and Jasinski, Donna |
| A comparison of Internet usage between two residency programs in the United Kingdom and the United States | | 2002 | Journal of Clinical Anesthesia | 14 | 388-94 | Sanders, J. C. |
| European Airway Management Society (EAMS) structure and activities | | 2018 | Trends in Anaesthesia and Critical Care | 18 | 13-15 | Saracoglu, K. T. and Sorbello, M. and Saracoglu, A. |
| Effective communication of difficult airway management to subsequent anesthesia providers | | 2009 | Anesthesia & Analgesia | 109 | 684-6 | Schaeuble, J. C. and Caldwell, J. E. |
| Measurement of Information Transfer During Simulated Sequential Complete Shift-to-Shift Intraoperative Handoffs. Mayo Clinic Proceedings: Innovations, Quality & Outcomes. 2023 Feb 1;7(1):9-19. | |  |  |  |  | Schiavi A, Mershon BH, Gottschalk A, Miller CR. |
| Assessing electronic interruptions experienced by an anesthesiology clinical director | | 2016 | Journal of Clinical Anesthesia | 34 | 658-60 | Schulte, T. E. and Roberts, E. K. and Birch, K. and Lisco, S. J. |
| Coaching for the pediatric anesthesiologist: becoming our best selves. Paediatr Anaesth. 2021;31:85–91 | |  |  |  |  | Schwartz JM, Wittkugel E, Markowitz SD, Lee JK, Deutsch N. |
| Empowering Women as Leaders in Pediatric Anesthesiology: Methodology, Lessons, and Early Outcomes of a National Initiative | | 2021 | Anesthesia & Analgesia | 133 | 1497-1509 | Schwartz, J. M. and Markowitz, S. D. and Yanofsky, S. D. and Tackett, S. and Berenstain, L. K. and Schwartz, L. I. and Flick, R. and Heitmiller, E. and Fiadjoe, J. and Lee, H. H. and Honkanen, A. and Malviya, S. and Cladis, F. P. and Lee, J. K. and Deutsch, N. |
| How social media is changing the practice of regional anesthesiology. Curr Anesthesiol Rep 2017;7:238-45. | |  |  |  |  | Schwenk ES, Chu LF, Gupta RK, Mariano ER. |
| I Tweet, therefore I learn: an analysis of Twitter use across anesthesiology conferences. Anesthesia & Analgesia. 2020 Feb 1;130(2):333-40. | |  |  |  |  | Schwenk ES, Jaremko KM, Park BH, Stiegler MA, Gamble JG, Chu LF, Utengen A, Mariano ER. |
| Development, Implementation, and Evaluation of a Certified Registered Nurse Anesthetist Preceptorship-Mentorship Program | | 2017 | Journal of Continuing Education in Nursing | 48 | 464-473 | Scott-Herring, M. and Singh, S. |
| A Distance Blended Learning Program to Upgrade the Clinical Competence of District Non-doctor Anesthesia Providers in Nepal | | 2017 | World Journal of Surgery | 41 | 3006-3011 | Shah, S. and Knoble, S. and Ross, O. and Pickering, S. |
| Distance-Learning Initiatives Targeting Non-physician Anesthesia Providers in Low-Resource Environments | | 2021 | Current Anesthesiology Reports | 11 | 64-68 | Shah, S. and Ross, O. and Pickering, S. |
| Tablet e-Logbooks: Four Thousand Clinical Cases and Complications e-Logged by 14 Nondoctor Anesthesia Providers in Nepal | | 2017 | Anesthesia & Analgesia | 125 | 1337-1341 | Shah, S. and Ross, O. and Pickering, S. and Knoble, S. and Rai, I. |
| Multimedia training support for anaesthetists | | 2017 | The clinical teacher | 14 | 68-70 | Shanahan, E. and Kearney, M. |
| Involving users in the design of a system for sharing lessons from adverse incidents in anaesthesia | | 2006 | Anaesthesia | 61 | 350-4 | Sharma, S. and Smith, A. F. and Rooksby, J. and Gerry, B. |
| What makes for good anesthesia teaching by faculty in the operating room? The perspective of anesthesiology residents. Cureus. 2018;10(5). | |  |  |  |  | Shin W, Tanaka P, Rafael V, Marty A, Macario A. |
| Developing and validating a tool for measuring the educational environment in clinical anesthesia. Canadian Journal of Anesthesia. 2018 Nov 1;65(11):1228-39. | |  |  |  |  | Sidhu NS, Clissold E. |
| Improving nurse anesthetist intraoperative handoff process by developing and implementing an evidence-based, facility-specific cognitive aid. Journal of Nursing & Interprofessional Leadership in Quality & Safety. 2019;2(2):1. | |  |  |  |  | Silva J, Arnaud M. |
| A Critical analysis of anesthesiology podcasts: identifying determinants of success. JMIR Med Educ. 2016;2:e14. | |  |  |  |  | Singh D, Alam F, Matava C. |
| In Response to ‘Anaesthesiologist and social media: Walking the fine line’. Indian Journal of Anaesthesia. 2019 Mar 1;63(3):253-4. | |  |  |  |  | Singh SK, Mistry T. |
| Peer support in anaesthesia: development and implementation of a peer-support programme within the Royal Brisbane and Women’s Hospital Department of Anaesthesia and Perioperative Medicine. Anaesth Intensive Care 2019;47: 497–502 | |  |  |  |  | Slykerman G, Wiemers MJ, Wyssusek KH. |
| ‘Help! I need somebody’: getting timely assistance in clinical practice. Anaesthesia. 2016 Jul;71(7):755-9. | |  |  |  |  | Smith MA, Byrne AJ. |
| That's your patient. There's your ventilator': exploring induction to work experiences in a group of non-UK EEA trained anaesthetists in a London hospital: a qualitative study | | 2015 | BMC Medical Education | 15 | 50 | Snelgrove, H. and Kuybida, Y. and Fleet, M. and McAnulty, G. |
| Stress and burnout in anesthesia residency: an exploratory case study of peer support groups. Qualitative Research in Medicine and Healthcare. 2018 Sep 3;2(2). | |  |  |  |  | Spence J, Smith D, Wong A. |
| Comparison between High-and Low-Cost Transmission of Tele-Anesthesia in Japan. Journal of Healthcare Engineering. 2018 Jun 11;2018. | |  |  |  |  | Sugawara Y, Miyashita T, Mizuno Y, Nagamine Y, Miyazaki T, Kobayashi A, Tojo K, Iketani Y, Takaki S, Goto T. |
| Rapid Debriefings Following Critical Incidents in Pediatric Anesthesiology. Pediatric Anesthesia. 2022 Dec 20. | |  |  |  |  | Sullivan L, Park R, Staffa SJ, Cravero J, Vinson AE. |
| Association between handover of anesthesiology care and 1-year mortality among adults undergoing cardiac surgery. JAMA Network Open. 2022 Feb 1;5(2):e2148161-. | |  |  |  |  | Sun LY, Jones PM, Wijeysundera DN, Mamas MA, Eddeen AB, O’Connor J. |
| Challenging authority during a life-threatening crisis: the effect of operating theatre hierarchy | | 2013 | British Journal of Anaesthesia | 110 | 463-71 | Sydor, D. T. and Bould, M. D. and Naik, V. N. and Burjorjee, J. and Arzola, C. and Hayter, M. and Friedman, Z. |
| Intraoperative handoffs. International anesthesiology clinics. 2013 Jan 1;51(1):31-42. | |  |  |  |  | Tan JA, Helsten D. |
| Implementation of a Needs-Based, Online Feedback Tool for Anesthesia Residents With Subsequent Mapping of the Feedback to the ACGME Milestones | | 2017 | Anesthesia & Analgesia | 124 | 627-635 | Tanaka, P. and Bereknyei Merrell, S. and Walker, K. and Zocca, J. and Scotto, L. and Bogetz, A. L. and Macario, A. |
| Attitudes toward physician-nurse collaboration in anesthesia | | 2009 | AANA Journal | 77 | 343-348 | Taylor, C. L. |
| Intraoperative care transitions are not associated with postoperative adverse outcomes. Anesthesiology. 2016 Oct;125(4):690-9. | |  |  |  |  | Terekhov MA, Ehrenfeld JM, Dutton RP, Guillamondegui OD, Martin BJ, Wanderer JP. |
| Understanding the perceptions and experiences of Certified Registered Nurse Anaesthetists regarding handovers: a focus group study | | 2014 | Nursing open | 1 | 32-41 | Testa, Denise and Emery, Susan |
| Implementation of a certified registered nurse anesthetist second victim peer support program | | 2022 | Journal of Perianesthesia Nursing | 37 | 167-173. e1 | Thompson, Mallory and Hunnicutt, Richard and Broadhead, Marleigh and Vining, Brooke and Aroke, Edwin N |
| Social network analysis and quantification of a prototypical acute pain medicine and regional anesthesia service. Pain Medicine. 2012 Jun 1;13(6):808-19. | |  |  |  |  | Tighe PJ, Smith JC, Boezaart AP, Lucas SD. |
| Perceptions of Certified Registered Nurse Anesthetists on Factors Affecting Their Transition From Student | | 2017 | AANA Journal | 85 | 438-444 | Tracy, Andy |
| Social media for the regional anesthesiologist: can we use it in place of medical journals? | | 2020 | Regional Anesthesia & Pain Medicine | 45 | 239-242 | Tran, B. W. and Dhillon, S. K. and Overholt, A. R. and Huntoon, M. |
| An anesthesia preinduction checklist to improve information exchange, knowledge of critical information, perception of safety, and possibly perception of teamwork in anesthesia teams. Anesthesia & Analgesia. 2015 Oct 1;121(4):948-56. | |  |  |  |  | Tscholl DW, Weiss M, Kolbe M, Staender S, Seifert B, Landert D, Grande B, Spahn DR, Noethiger CB. |
| Establishing an Anaesthesia and Intensive Care partnership and aiming for national impact in Tanzania | | 2016 | Global Health | 12 | 7 | Ulisubisya, M. and Jornvall, H. and Irestedt, L. and Baker, T. |
| Peer support in anesthesia: turning war stories into wellness. Curr Opin Anaesthesiol. 2018;31:382–387. | |  |  |  |  | Vinson AE, Randel G. |
| Assessing levels of support for residents following adverse outcomes: a national survey of anesthesia residency programs in the United States | | 2014 | Medical Teacher | 36 | 858-66 | Vinson, A. E. and Mitchell, J. D. |
| Anesthesia for liver transplantation in US academic centers: institutional structure and perioperative care | | 2012 | Liver Transplantation | 18 | 737-43 | Walia, A. and M and ell, M. S. and Mercaldo, N. and Michaels, D. and Robertson, A. and Banerjee, A. and Pai, R. and Klinck, J. and Weinger, M. and P and harip and e, P. and Schumann, R. |
| Impact of Mentorship, by Gender, on Career Trajectory in an Academic Anesthesiology Department: A Survey Study. Journal of Continuing Education in the Health Professions. 2022 Jan 1;42(1):14-8. | |  |  |  |  | Warner LL, Gali B, Oxentenko AS, Schroeder DR, Arendt KW, Moeschler SM. |
| Click for Help: An Anesthesiology Department Messaging System. Journal of Medical Systems. 2021 Jun;45(6):68. | |  |  |  |  | Wax DB, Adeel A, Huang J, Villar J, Levin MA. |
| Characteristics of emergency pages using a computer-based anesthesiology paging system in children and adults undergoing procedures at a tertiary care medical center. Anesthesia & Analgesia. 2013 Apr 1;116(4):904-10. | |  |  |  |  | Weingarten TN, Abenstein JP, Dutton CH, Kohn MA, Lee EA, Mullenbach TE, Narr BJ, Schroeder DR, Sprung J. |
| Lasting impact: insights from a surgical mission-based mentoring training programme in the Republic of Congo. BMJ Global Health. 2016 Oct 1;1(3):e000102. | |  |  |  |  | White M, Close K. |
| Creation and evaluation of an anesthesiology and critical care podcast. The Journal of Education in Perioperative Medicine: JEPM. 2018 Jan;20(1). | |  |  |  |  | Wolpaw J, Toy S. |
| Do electronic mail discussion lists act as virtual colleagues? | | 1997 | Proceedings_OR_AMIA Annual Fall Symposium | | 325-9 | Worth, E. R. and Patrick, T. B. |
| Examining transfer of care processes in nurse anesthesia practice: introducing the patient protocol. AANA J. 2013;81(3):225-232 | |  |  |  |  | Wright SM. |
| Anaesthesia and the Internet | | 1996 | Anaesthesia | 51 | 677-82 | Yentis, S. M. and Ooi, R. |
| Medical resources for the anesthesiologist on the Internet | | 2000 | Regional Anesthesia & Pain Medicine | 25 | 99-102 | Zura, A. and Smith, M. P. |

* included as separate column if not already cited with title
