# Supplementary material for: Communication Between Anaesthesia Providers for Clinical and Professional Purposes: A Scoping Review
Source: Anesthesiol Res Pract. 2025 Mar 6;2025:3598234. doi: 10.1155/anrp/3598234 (PMC11991797; doi:10.1155/anrp/3598234)
Supplement: Supporting Information 5 — Supporting file 5: Articles excluded on the basis of language. [file 3598234.f5.docx]

**Supplementary file 5: Articles excluded on the basis of language**

| German | 31 |
| --- | --- |
| Spanish | 11 |
| Chinese | 3 |
| Hebrew | 2 |
| Italian | 2 |
| Japanese | 2 |
| Korean | 2 |
| Russian | 2 |
| Bosnian | 1 |
| Czech | 1 |
| Dutch | 1 |
| Greek | 1 |
| Persian | 1 |
| Polish | 1 |
| Thai | 1 |
| Turkish | 1 |
