# Supplementary material for: Communication Between Anaesthesia Providers for Clinical and Professional Purposes: A Scoping Review
Source: Anesthesiol Res Pract. 2025 Mar 6;2025:3598234. doi: 10.1155/anrp/3598234 (PMC11991797; doi:10.1155/anrp/3598234)
Supplement: Supporting Information 6 — Supporting file 6: Visual abstract. [file 3598234.f6.pdf]

Communication between anaesthesia providers: a scoping review

Inter-anaesthetist communication is relevant across a wide range of domains but is poorly described, particularly in low-resource settings

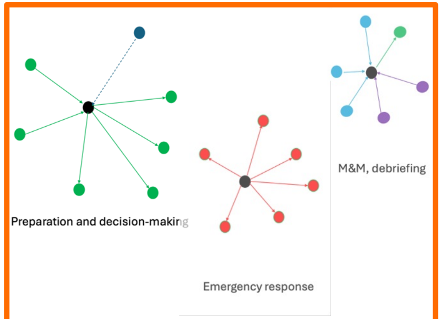

Anaesthesia providers need to communicate with one another for clinical and professional purposes: what is known about how this occurs?

- **Scoping review using JBI methodology**
- **Search strategy foci:**
  - networks
  - communities of practice
  - interprofessional working
- **Literature mapped by**
  - provider cadre
  - location
  - modality of communication
  - purpose &/ outcome of communication

Findings:

Retrieved: 3872 records

Included: 225 records

Purpose of communication:

- **clinical output 49.3%**
  … handover, NTS, advice, coordination…
- **professional activity 24.4%**
  … training, supervising, evaluation…
- **wellbeing/development 26.2%**
  … mentoring, debriefing, support…

Providers:

- **physicians 81% (29% with nurses)**
- **nurses 37% (29% with physicians)**
- **not stated 11%**

Locations:

- **HICs: 72%**
- **LMICs: 6.2%**
- **mixed: 6.2%**
- **unstated: 15.5%**

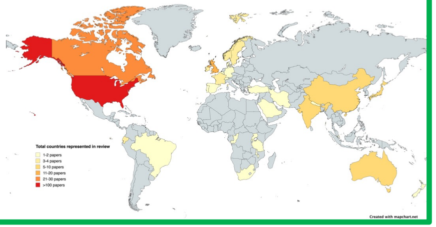

H Edgcombe, G Murithi, M Manyano, S Dunin, N Thurley, H Higham, M English, C Blacklock
